# Molecular Evolutionary Analyses of the Spike Protein Gene and Spike Protein in the SARS-CoV-2 Omicron Subvariants

**DOI:** 10.3390/microorganisms11092336

**Published:** 2023-09-18

**Authors:** Norika Nagasawa, Ryusuke Kimura, Mao Akagawa, Tatsuya Shirai, Mitsuru Sada, Kaori Okayama, Yuka Sato-Fujimoto, Makoto Saito, Mayumi Kondo, Kazuhiko Katayama, Akihide Ryo, Makoto Kuroda, Hirokazu Kimura

**Affiliations:** 1 Department of Health Science, Gunma Paz University Graduate School of Health Sciences, 1-7-1, Tonya-machi, Takasaki-shi 370-0006, Gunma, Japan; nagasawa@paz.ac.jp (N.N.); okayama@paz.ac.jp (K.O.); 2 Department of Medical Technology, Gunma Paz University School of Medical Science and Technology, 1-7-1, Tonya-machi, Takasaki-shi 370-0006, Gunma, Japan; fujimoto@paz.ac.jp; 3 Advanced Medical Science Research Center, Gunma Paz University Research Institute, 1338-4, Shibukawa, Shibukawa-shi 377-0008, Gunma, Japan; m2220015@gunma-u.ac.jp (R.K.); shirait28@gmail.com (T.S.); 4 Department of Bacteriology, Gunma University Graduate School of Medicine, Maebashi-shi 371-8514, Gunma, Japan; 5 Department of Clinical Laboratory, Juntendo University Hospital, Bunkyo-ku, Tokyo 113-8431, Japan; akagawa6313@gmail.com; 6 Department of Respiratory Medicine, Kyourin University School of Medicine, 6-20-2, Shinkawa, Mitaka-shi 181-8611, Tokyo, Japan; rainbow_orchestra716@yahoo.co.jp; 7 Department of Clinical Engineering, Gunma Paz University School of Medical Science and Technology, Takasaki-shi 370-0006, Gunma, Japan; ma-saito@paz.ac.jp (M.S.); kondo@paz.ac.jp (M.K.); 8 Laboratory of Viral Infection Control, Ōmura Satoshi Memorial Institute, Graduate School of Infection Control Sciences, Kitasato University, 5-9-1, Shirogane, Minato-ku, Tokyo 108-8641, Japan; katayama@lisci.kitasato-u.ac.jp; 9 Department of Virology III, National Institute of Infectious Diseases, 4-7-1, Gakuen, Musashimurayama-shi 208-0011, Tokyo, Japan; aryo@niid.go.jp; 10 Pathogen Genomics Center, National Institute of Infectious Diseases, 1-23-1, Toyama, Shinjuku-ku, Tokyo 162-8640, Japan; makokuro@niid.go.jp

**Keywords:** SARS-CoV-2 Omicron subvariants, evolution, spike protein gene, conformational epitope, receptor-binding domain (RBD), vaccine evasion

## Abstract

To better understand the evolution of the SARS-CoV-2 Omicron subvariants, we performed molecular evolutionary analyses of the spike (*S*) protein gene/S protein using advanced bioinformatics technologies. First, time-scaled phylogenetic analysis estimated that a common ancestor of the Wuhan, Alpha, Beta, Delta variants, and Omicron variants/subvariants diverged in May 2020. After that, a common ancestor of the Omicron variant generated various Omicron subvariants over one year. Furthermore, a chimeric virus between the BM.1.1.1 and BJ.1 subvariants, known as XBB, diverged in July 2021, leading to the emergence of the prevalent subvariants XBB.1.5 and XBB.1.16. Next, similarity plot (SimPlot) data estimated that the recombination point (breakpoint) corresponded to nucleotide position 1373. As a result, XBB.1.5 subvariants had the 5′ nucleotide side from the breakpoint as a strain with a BJ.1 sequence and the 3′ nucleotide side as a strain with a BM.1.1.1 sequence. Genome network data showed that Omicron subvariants were genetically linked with the common ancestors of the Wuhan and Delta variants, resulting in many amino acid mutations. Selective pressure analysis estimated that the prevalent subvariants, XBB.1.5 and XBB.1.16, had specific amino acid mutations, such as V445P, G446S, N460K, and F486P, located in the RBD when compared with the BA.4 and BA.5 subvariants. Moreover, some representative immunogenicity-associated amino acid mutations, including L452R, F486V, R493Q, and V490S, were also found in these subvariants. These substitutions were involved in the conformational epitopes, implying that these mutations affect immunogenicity and vaccine evasion. Furthermore, these mutations were identified as positive selection sites. These results suggest that the *S* gene/S protein Omicron subvariants rapidly evolved, and mutations observed in the conformational epitopes may reduce the effectiveness of the current vaccine, including bivalent vaccines such as mRNA vaccines containing the BA.4/BA.5 subvariants.

## 1. Introduction

A new coronavirus disease 2019 (COVID-19) caused by the severe acute respiratory syndrome coronavirus type 2 (SARS-CoV-2) suddenly emerged and became a pandemic from 2020 to early 2023. Although on 5 May 2023, the World Health Organization (WHO) lifted the declaration of a Public Health Emergency of International Concern, the virus is still endemic in many countries [1,2].

SARS-CoV-2 has been generating many variants and subvariants. These variants/subvariants have been associated with prevalent surges [1,2,3]. For example, the Alpha (α) variant was prevalent in late 2020 [4]. The Delta (δ) variant was also prevalent in 2021 [4]. After that, the Omicron (ο) variant emerged and became prevalent after late 2021 [4]. The Omicron variants further generated various subvariants, such as BA.1, BA.2, BA.4, and BA.5, and these subvariants caused large epidemics [4]. Moreover, the BA.2 subvariants recombined between the BJ.1 subvariant and the BM.1.1.1 subvariant, resulting in a new subvariant, XBB [4]. Currently (June 2023), the recombinants XBB.1.5 and XBB.1.16 are reportedly prevalent subvariants in many countries, including Japan [4].

Some vaccines, such as mRNA-type vaccines against SARS-CoV-2, have been developed, and these vaccines may be highly effective in preventing disease onset and/or aggravation [5,6,7]. An aggressive vaccine campaign may be a major factor in the suppression and control of COVID-19 [8].

The spike (S) protein of SARS-CoV-2 is not only a major antigen but also the target of vaccines. It is suggested that the S protein gene has been evolving, leading to alterations in its infectivity and antigenicity [9]. The alteration of the antigenicity of the protein may affect the efficacy of the vaccines. However, the changes accompanying the molecular evolution of the *S* gene and S protein are not exactly known.

Conformational epitopes may be linked to the binding sites of neutralizing antibodies (NT-Ab) [10]. If there are many amino acid mutations in the antigen, including the S protein of the epitope motifs, the effectiveness of the NT-Ab against the epitopes may be reduced. Therefore, predicting the epitopes and their amino acid mutations may be useful for predicting vaccine effects [10,11,12,13]. Currently, some mRNA vaccine agents against SARS-CoV-2 are formulated with the sequences of the BA.4/BA.5 subvariants [13]. Therefore, in this study, to elucidate the phylogeny, molecular evolutionary processes, and antigenic changes in the *S* gene/S proteins, we comprehensively performed molecular evolutionary analyses of them in various Omicron subvariants using advanced and authentic bioinformatics technologies. Furthermore, based on these results, we predicted the efficacy of mRNA vaccines against SARS-CoV-2. We report our findings here.

## 2. Materials and Methods

### 2.1. Strain Selection

Full-length nucleotide sequences of the SARS-CoV-2 reference strain and 16 representative strains with major mutations were downloaded from NCBI [https://www.ncbi.nlm.nih.gov/ (accessed on 10 August 2023)] and GISIDE [https://gisaid.org/ (accessed on 10 August 2023)]. The representative strains were determined based on the oldest collection dates, except for strains with no information on the region and year of detection or isolation and ambiguous sequences. The sequences used for each analysis are as follows: Wuhan, NCBI MN908947 (SARS-CoV-2 reference strain); Alpha, GISIDE EPI_ISL_878492; Beta, GISIDE EPI_ISL_1007659; Delta, GISIDE EPI_ISL_2038893; BA.1, GISIDE EPI_ISL_7988149; BA.2, GISIDE EPI_ISL_9028491; BA.2.10, GISIDE EPI_ISL_11520937; BA.2.75, GISIDE EPI_ISL_13302209; BM.1.1.1, NCBI OX361494; BA.4, GISIDE EPI_ISL_13389730; BA.5, GISIDE EPI_ISL_14243867; BQ.1, GISIDE EPI_ISL_14333750; BJ.1, NCBI OX339969; XBB, GISIDE EPI_ISL_16338736; XBB.1.5, GISIDE EPI_ISL_16071348; XBB.1.16, GISIDE EPI_ISL_17620500. After multiple alignment using MAFFT Version 7 [14], amino acid sequences other than S protein were deleted, and the S protein amino acid sequence of each SARS-CoV-2 mutation was used as the dataset.

### 2.2. Time-Scaled Phylogenetic Analysis

To construct a phylogenetic tree of the *S* gene in all SARS-CoV-2 variants/subvariants and estimate its evolutionary rate, we used the Bayesian Markov Chain Monte Carlo (BMCMC) method from BEAST Version 2.6.6 [15]. First, the jModelTest 2.1.10 program was used to determine the optimal substitution model (HKY+I) [16]. Next, the path sampling/stepping-stone sampling method was used to search for the best of the four clock models (Strict Clock, Relaxed Clock Exponential, Relaxed Clock Log Normal, and Random Local Clock) and two prior tree models (Coalescent Constant Population and Coalescent Exponential Population). The optimal dataset was estimated to be the Random Local Clock model and the Coalescent Exponential Population model. The MCMC chain was run for 100,000,000 steps and sampled every 10,000 steps. Convergence was evaluated by effective sample size using Tracer Version 1.7.2 [17], and values above 200 were considered acceptable. FigTree v1.4.4 was used to illustrate the phylogenetic tree. Tracer Version 1.7.2 was used to estimate the evolutionary rate of SARS-CoV-2. Moreover, we also calculated the evolutionary rate of the SARS-CoV-2 strains, excepting all Omicron variants/subvariants strains. Similarly, we also estimated the evolutionary rate of the recombinant strains including XBB, XBB.1.5, and XBB.1.16.

### 2.3. Statistical Analysis

We used the Kruskal–Wallis and Bonferroni tests in the EZR statistical software (Version 1.61) to compare the evolutionary rates among the *S* genes in SARS-CoV-2 variants/subvariants [18]. *p*-values of less than 0.05 were considered statistically significant.

### 2.4. Similarity Plot (SimPlot) Analysis

The nucleotide sequence similarity of the representative strains of BA.2, BJ.1, BM.1.1.1, XBB.1.5, and XBB.1.16 was calculated using SimPlot software (version 3.5.1). The sequence of BA.2 reference strain (GISIDE EPI_ISL_9028491) was used as the query sequence. The similarity was examined using the Kimura 2-parameter method with a window size of 200 nucleotides in length and a step size of 20 nucleotides in the *S* gene.

### 2.5. Genome Network Analysis

To evaluate the haplotype network, the median-joining network was drawn using PopArt version 1.7 software. The nucleotide sequences of 16 strains were used as the dataset, and the tolerance parameter (Epsilon) was set to 0.

### 2.6. Homology Modeling

The three-dimensional (3D) structures of BA.2, BA.4, BA.5, XBB.1.5, XBB.1.16, BA.2.10, BA.2.75, BJ.1, and BM.1.1.1 S proteins were not available. Thus, we constructed them using homology modeling. First, we downloaded the crystal structure of SARS-CoV-2 trimeric S protein (PDBID: 6XR8) from the Protein Data Bank [https://www.rcsb.org/ (accessed on 2 June 2023)] as the template for homology modeling. Next, we identified the amino acid mutations in each subvariant S protein in comparison to the template based on previous reports [19,20,21]. Then, we created amino acid-substituted S proteins using MODELLER 10.1 [22]. The optimal models for each subvariant were selected by evaluating the structure reliability through Ramachandran plot analysis in WinCoot v.0.9.4.1 [23]. The selected models were energetically minimized using GROMOS96 implemented in the Swiss-PDB viewer software v4.1.0 [24].

### 2.7. Conformational Epitope Analysis

Conformational epitope analysis was performed to clarify the epitope changes between the constructed models. In this analysis, we used the monomer from the trimeric S protein. Conformational epitope analysis of these proteins was performed using SEMA, DiscoTope-2.0, SEPPA-3.0, and ElliPro with cutoff values set at 0.76 (SEMA), −3.7 (DiscoTope-2.0), 0.1 (SEPPA-3.0), and 0.5 (ElliPro) based on previous reports [25,26,27,28,29]. Then, regions predicted to be epitopes by four or more of these methods and having three or more contiguous amino acid residues were determined as conformational epitopes.

### 2.8. Selective Pressure Analysis

We performed a selection analysis to assess the relationships among the selection pressure sites, receptor-binding domain (RBD), and amino acid mutations. The full-length SARS-CoV-2 viral nucleotides of BA.2, BA.4, BA.5, XBB.1.5, and XBB.1.16 were randomly collected from Nextstrain [https://nextstrain.org/ (accessed on 10 August 2023)] and GISIDE for 25 strains each between April 2022 and April 2023. Alignment was performed using MAFFT v7, and only the target S protein was extracted. Strains with large sequence defects or unknown genome sequences were deleted, and the final dataset consisted of 102 strains. The selection analysis was performed using the internal fixed-effects likelihood (IFEL) using the Datamonkey server [https://www.datamonkey.org/ (accessed on 10 September 2023)] [30]. The cutoff *p*-value was set at 0.05.

### 2.9. Visualizing Epitopes on 3D Structure Model of SARS-CoV-2 Spike Protein

We visualized the 3D structures of the SARS-CoV-2 S proteins using PyMOL software v2.3.4 [31]. Moreover, we mapped the predicted conformational epitopes, the selection pressure sites, RBD, and previously reported main mutation sites [19,20,21] on the S proteins.

## 3. Results

### 3.1. Time-Scaled Phylogeny of S Gene in the Various SARS-CoV-2 Variants/Subvariants

**Figure 1:**
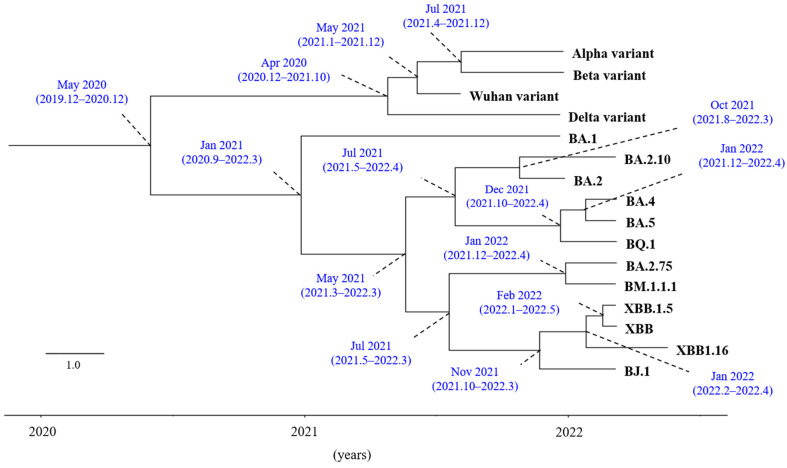
Time-scaled phylogenetic tree based on the full-length *S* gene nucleotide sequences using Bayesian MCMC (BMCMC) method. Maximum clade credibility tree from a dataset of SARS-CoV-2 variants *S* gene. Each mutation’s first branch point (node) is represented by a plot, and parentheses indicate 95% HPDs.

To estimate the time-scaled phylogeny of the *S* gene in SARS-CoV-2 variants/subvariants, we constructed a phylogenetic tree using the BMCMC method. As shown in Figure 1, a common ancestor of Omicron variants and other SARS-CoV-2 strains diverged in May 2020. Afterward, a common ancestor of the Omicron variants further diverged into Omicron subvariants, including BA.1, BA.2, BA.4, and BA.5, over the course of one year. Additionally, a common ancestor of Omicron XBB, BM.1.1.1, and BJ.1 emerged in July 2021. Next, the mean evolutionary rate of the *S* gene in all SARS-CoV-2 variants/subvariants was estimated to be 7.77 × 10^−4^ substitutions/site/year (s/s/y) (95% HPD, 2.74 × 10^−4^ to 1.31 × 10^−3^ s/s/y). The mean estimated evolutionary rate of the Omicron variants/subvariants was calculated to be 3.43 × 10^−3^ substitutions/site/year (s/s/y) (95% HPD, 2.16 × 10^−4^ to 8.54 × 10^−3^ s/s/y). The rates of the Wuhan, Alpha, Beta, and Delta strains were estimated to be 8.25 × 10^−4^ substitutions/site/year (s/s/y) (95% HPD, 2.356 × 10^−4^ to 1.509 × 10^−3^ s/s/y). Moreover, the rates of the recombinants, including XBB, XBB.1.5, and XBB.1.16, were estimated to be 1.188 × 10^−3^ substitutions/site/year (s/s/y) (95% HPD, 2.0576 × 10^−5^ to 1.6975 × 10^−3^ s/s/y). No statistical significance was found among these rates. These results suggest that Omicron variants/subvariants evolved relatively rapidly and generated various other subvariants within only one year.

### 3.2. Similarity Plot Analysis of Omicron Subvariants BA.2, BJ.1, BM.1.1.1, XBB.1.5, and XBB.1.16

**Figure 2:**
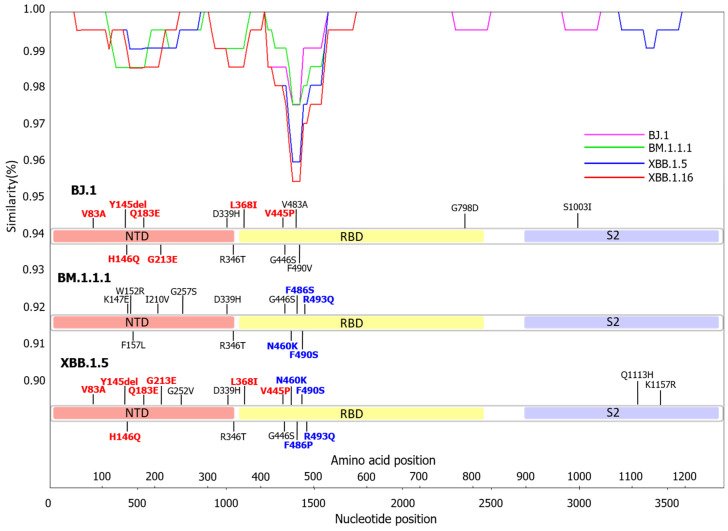
Similarity plot analysis of SARS-CoV-2 Omicron subvariants were conducted. The results of the SimPlot analysis are shown in pink for BJ.1, green for BM.1.1.1, blue for XBB.1.5, and red for XBB.1.16. Nucleotide sequences of the BA.2, BJ.1, BM.1.1.1, and XBB.1.5 strains used in the analysis were compared. The mutations that match between BJ.1 and XBB.1.5 are shown in red. The mutations that match between BM.1.1.1 and XBB.1.5 are shown in blue.

We performed recombination analysis of the *S* gene between Omicron subvariants BJ.1, BM.1.1.1, and XBB.1.5. Figure 2 reveals that the nucleotide identities among them significantly fluctuated at specific nucleotide positions, particularly around position number 1373 (Figure 2). The nucleotide identities between BJ.1 and XBB.1.5 before the breakpoint were high, while the identities between BM.1.1.1 and XBB.1.5 after the breakpoint were also high. The SimPlot data also estimated that the recombination point (breakpoint) corresponded to the nucleotide position 1373. As a result, XBB.1.5 subvariants had the 5′ nucleotide side from the breakpoint as a strain with a BJ.1 sequence and the 3′ nucleotide side as a strain with a BM.1.1.1 sequence. Moreover, amino acid mutations in the S protein of XBB.1.5 almost completely matched with the parent strains, BJ.1 and BM.1.1.1. The results suggest that the XBB.1.5 subvariant was a recombinant between BJ.1 and BM.1.1.1 subvariants.

### 3.3. Genome Network Analysis Based on the S Gene Sequences

**Figure 3:**
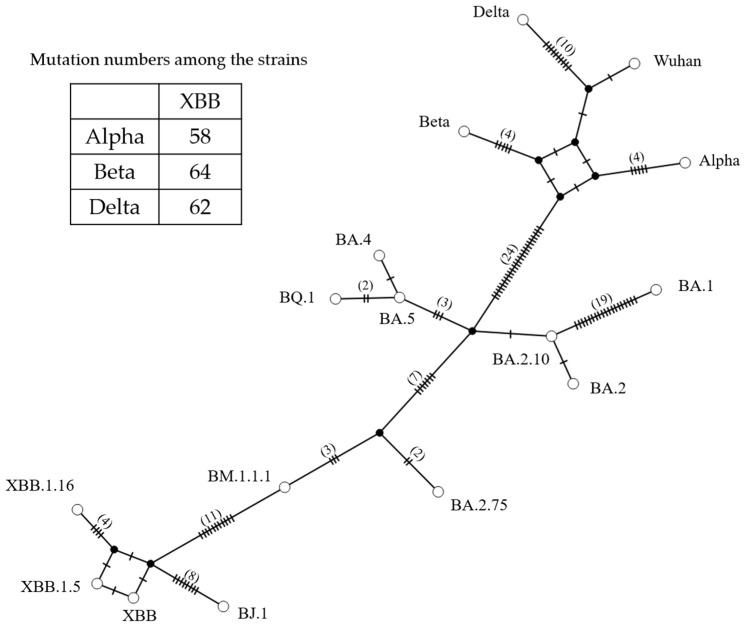
SARS-CoV-2 variant *S* gene constructed using genome network analysis. Each circle represents a haplotype, and the number of branch bars connecting the haplotypes and the number in parentheses represents the number of different bases between haplotypes. The numbers in the table of this figure indicate the numbers of nucleotide substitutions among the strains.

We also analyzed the genome network among the *S* genes in the SARS-CoV-2 variants/subvariants (Figure 3). Initially, an ancestor of the Omicron BA subvariant formed a genetic network with the Alpha variant through 24 nucleotide substitutions. Additionally, the ancestor of the Omicron BA subvariant also established a genetic network with the BA.2.75 subvariant through seven nucleotide substitutions. Furthermore, an ancestor of the XBB subvariant formed genetic networks with the BJ.1 and BM.1.1.1 subvariants through 7 and 11 nucleotide substitutions, respectively. The numbers of nucleotide substitutions between the *S* gene in the XBB and Alpha, Beta, and Delta were 58, 64, and 62, respectively. These results suggest that the Omicron XBB subvariant evolved by acquiring numerous nucleotide mutations from other variants, such as Alpha, Beta, and Delta, which acted as parental variants.

### 3.4. Mapping of the Conformational Epitopes and Amino Acid Mutations

**Figure 4:**
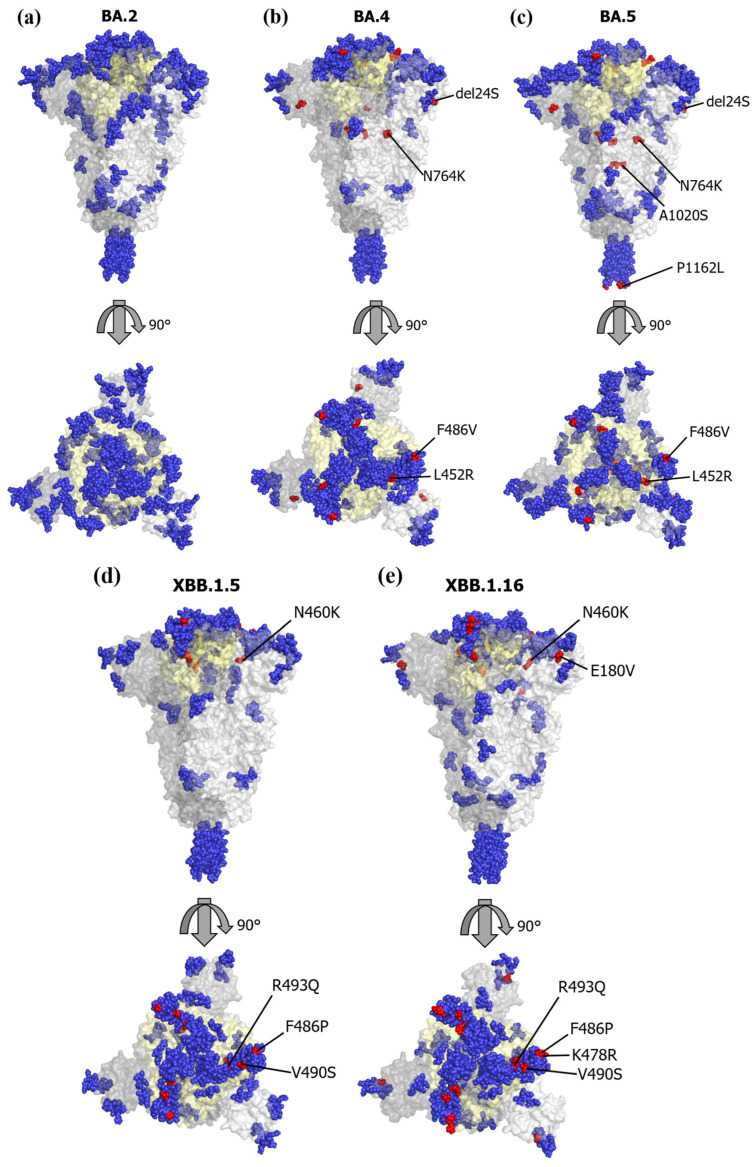
Conformational epitopes and main mutations mapped on three-dimensional structure models of SARS-CoV-2 S protein. We mapped the predicted conformational epitopes on three-dimensional structure models of SARS-CoV-2 S protein, including the receptor-binding domain (RBD). The chains of the trimer structures are colored in dark gray (chain A), gray (chain B), and light gray (chain C). Conformational epitopes, main mutation sites, and RBD are shown in blue, red, and light-yellow colors, respectively. (**a**) SARS-CoV-2 BA.2 variant S protein 3D structure; (**b**) SARS-CoV-2 BA.4 variant S protein 3D structure; (**c**) SARS-CoV-2 BA.5 variant S protein 3D structure; (**d**) SARS-CoV-2 XBB.1.5 variant S protein 3D structure; (**e**) SARS-CoV-2 XBB.1.16 variant S protein 3D structure.

We mapped the conformational epitopes and amino acid mutations in BA.4, BA.5, XBB.1.5, and XBB.1.16 on the three-dimensional (3D) structure of the S proteins (Figure 4a–e). We also mapped amino acid mutations referring to a prototype of the BA.2 subvariant. Firstly, most conformational epitopes of all subvariants were located in the RBD. The number of epitopes was 11 in the XBB.1.5 and XBB.1.16 subvariants and 12 in the BA.2, BA.4, and BA.5 subvariants (Figure S1). Among these epitopes, common amino acid motifs were found. Notably, the XBB.1.5 and XBB.1.16 subvariants possessed specific amino acid mutations, such as V445P, G446S, N460K, and F486P, which were located in the RBD when compared with the BA.4 and BA.5 subvariants. Moreover, some representative immunogenicity-associated amino acid mutations, such as L452R, F486V, R493Q, and V490S, were found in these subvariants. These substitutions were involved in the conformational epitopes (Table 1), suggesting that these substitutions may reflect the immunogenicity and vaccine evasion. We also examined the relationships between 3D structures and conformational epitopes among BA.2, BA.2.10, and BA.2.75 (Figure S1). As a result, we found that the length of the epitope motifs between BA.2 and BA.2.10 were partly changed, while amino acid mutations did not change. However, four amino acid mutations between BA.2 and BA.2.75 were found. These substitutions also overlapped with the conformational epitopes.

Most conformational epitopes in BJ.1 and BM.1.1.1 were located in the RBD. The number of epitopes was 14 in BJ.1 and 17 in BM.1.1.1 (Figure S1). In particular, BJ.1 had a similar amino acid substitution of V445P in the RBD, and BM.1.1.1 had a similar amino acid substitution of N460K, comparing with XBB.1.5 and XBB.1.16 (Table 2). These results suggest that there is no significant antigenic variation between the BJ.1, BM.1.1.1, and XBB strains.

### 3.5. Selective Pressure Analysis

We also performed selective pressure analysis on these amino acid mutations using the IFEL method (Table 3). As a result, many amino acid mutations were estimated as positive selection sites, with most of them located in the RBD. No negative selection site was found in the S protein. These results suggest that the amino acid mutations found in these subvariants may act as immune escape mechanisms against host defense.

## 4. Discussion

In this study, we performed evolutionary analyses of the *S* gene/S protein in the SARS-CoV-2 Omicron subvariants. First, a time-scaled phylogenetic tree suggested that a common ancestor of Omicron variants and other SARS-CoV-2 strains diverged in May 2020. Furthermore, a common ancestor of Omicron XBB, BM.1.1.1, and BJ.1 emerged in July 2021 (Figure 1). Second, SimPlot analysis suggested that the XBB subvariant was a recombinant between the BJ.1 and BM.1.1.1 subvariants (Figure 2). Next, the XBB subvariant evolved by acquiring many amino acid mutations from other variants, such as the Alpha, Beta, and Delta variants. Many amino acid mutations in the S protein of the XBB subvariant were involved in the conformational epitopes (Figure 4). Among them, some amino acid mutations were positively selected. These results suggest that the *S* gene/S protein in Omicron subvariants rapidly evolved with changes in immunogenicity, resulting in vaccine evasion.

First, to estimate the time-scaled phylogeny of the *S* gene in the SARS-CoV-2 variants/subvariants, we constructed a phylogenetic tree using the BMCMC method (Figure 1). We found that a common ancestor of Omicron variants diverged from a common ancestor of the Wuhan, Alpha, Beta, and Delta variants in May 2020. Subsequently, a common ancestor of the Omicron variants diverged into the Omicron subvariants, including BA.1, BA.2, BA.4, and BA.5, over one year. Furthermore, the emergence of a common ancestor of Omicron XBB, BM.1.1.1, and BJ.1 occurred in July 2021. The evolutionary rate of the Omicron variants/subvariants was estimated to be around 3.4 × 10^−3^ substitutions/site/year, which is similar to other rapidly evolving viruses such as the influenza virus and norovirus [32,33]. Moreover, we also assessed the evolutionary rates of the *S* gene in SARS-CoV-2 strains except for the Omicron subvariant strains. We also found that no significance of these rates of the *S* gene was found between the present SARS-CoV-2 strains. The results suggest that the Omicron subvariants’ *S* gene rapidly evolved and generated many further subvariants, such as the XBB subvariants, within only 1 year. These findings may be compatible with previous reports [3,34].

Next, it is suggested that the Omicron XBB subvariant was generated from the BJ.1 and BM.1.1.1 subvariants as a recombinant [35]. Thus, to estimate this, we performed a SimPlot analysis of the *S* gene with nucleotide substitutions among the BJ.1, BM.1.1.1, and XBB.1.5 subvariants (Figure 2). We discovered that nucleotide identities among them significantly changed at the breakpoint. Furthermore, nucleotide identities between BJ.1 and XBB.1.5 before the breakpoint were high, whereas the identities between BM.1.1.1 and XBB.1.5 after the breakpoint were high as well. Amino acid mutations in the S protein of XBB.1.5 almost completely matched with the parent strains (BJ.1 and BM.1.1.1). These results implicate that the XBB.1.5 subvariant is a recombinant between the BJ.1 and BM.1.1.1 subvariants, and these findings may be compatible with earlier reports [35].

We also constructed a genome network among various SARS-CoV-2 variants/subvariants based on the *S* gene sequences. We found that an ancestor of the Omicron BA subvariant formed a genetic network with the Alpha variant through many nucleotide substitutions (24 substitutions). The ancestor of the Omicron BA subvariant also formed a genetic network with the BA.2.75 subvariant through many nucleotide substitutions (7 substitutions). Moreover, an ancestor of the XBB subvariant formed a genetic network with the BJ.1 and BM.1.1.1 subvariants through many nucleotide substitutions (8 and 11 substitutions, respectively). These results indicate that the Omicron XBB subvariant evolved through many nucleotide substitutions in the *S* gene from parental variants such as the Alpha, Beta, and Delta variants.

Moreover, we studied the relationships between amino acid mutations and conformational epitopes in the S protein of various SARS-CoV-2 Omicron subvariants. Many conformational epitopes of all subvariants were located in the RBD. Within the epitopes, many common amino acid motifs were observed, although the locations of these epitopes on the RBD were distinct. Interestingly, there were some key amino acid mutations, such as V445P, G446S, N460K, and F486P, located in the RBD of the XBB.1.5 and XBB.1.16 subvariants, in comparison to the BA.4 and BA.5 subvariants. Additionally, we found representative immunogenicity-related amino acid mutations, including L452R, F486V, R493Q, and V490S, in these subvariants, encompassing the BA.4, BA.5, XBB.1.5, and XBB.1.16 subvariants. These mutations were identified as positive selection sites and were also located within the conformational epitopes. These findings suggest that these amino acid mutations may not only influence the immunogenicity of these subvariants but also contribute to the evasion of vaccine effects against the vaccine strains (Wuhan strain, a prototype, and BA.4 and BA.5 subvariants) [36,37].

The SARS-CoV-2 genome has been continuously evolving since its emergence, resulting in the generation of various subvariants. Until now, several variants, such as Alpha (α), Beta (β), Delta (δ), and Omicron (ο), have emerged and been associated with the pandemic [1,38]. Moreover, the Omicron variants have generated many subvariants, such as BA.1-5 and the XBB subvariants, which are the estimated progeny of BA.2 [18,20]. Currently, XBB.1.5 and XBB.1.16 are prevalent subvariants. Furthermore, these variants and subvariants exhibit many amino acid mutations in a major antigen, which is the S protein [20]. These mutations may be associated with changes in their antigenicity, leading to virus reinfection and/or vaccine evasion [37,39]. In general, positive selection sites may act as escape mutants in various viruses, including SARS-CoV-2 [40]. In the present study, many positive selection sites were found in the S proteins of the XBB.1.5 and XBB.1.16 subvariants (Table 2). Most of these sites were located in the conformational epitopes on the RBD (Figure 1 and Table 1). Moreover, the amino acid mutations between BA.2 and BA.2.75 also overlapped with the conformational epitopes. These amino acid mutations were also associated with changes in antigenicity and vaccine evasion [37]. These results suggest that these positive selection sites result in the evasion of host defense systems, such as immunity [37].

Next, there are two types of epitopes: linear and conformational epitopes [41]. Linear epitopes are continuous amino acid sequences of the primary amino acid sequences, while conformational epitopes are composed of discontinuous residues that are in proximity on the 3D protein structure. In general, these epitopes are recognized by the immune system, leading to the production of antibodies, including neutralizing antibodies (NT-Ab) [42]. It is suggested that over 90% of B-cell epitopes are conformational, while there are few linear epitopes [43]. Thus, it may be important to predict conformational epitopes in the antigen for estimating vaccine effects and reinfection by various pathogens [42]. Therefore, we mapped conformational epitopes and vaccine evasion-related amino acid mutations on the S proteins of various Omicron subvariants (Figure 4 and Table 1). As a result, many overlapping epitopes were found in these subvariants, along with some key amino acid mutations that may be associated with vaccine evasion against vaccine strains [44]. Indeed, there was a significant decline in NT-Ab titers against XBB.1.5 compared to the antibody titers in sera infected with the BA.4/BA.5 subvariants [45]. Our study showed that amino acid mutations in conformational epitopes within the RBD domains of the S protein in the XBB.1.5 and XBB.1.16 subvariants were consistent with the changes in the effects of neutralizing antibodies observed in previous in vitro studies [45]. To the best of our knowledge, this may be the first observation. Finally, we speculate as follows: the current vaccines containing BA.2 or BA.4/BA.5 subvariants may be effective in preventing aggravation due to XBB.1.5 and XBB.1.16 subvariant infections, although the onset prevention rates may be reduced due to the aforementioned key amino acid mutations [45]. Although these speculations should be validated through in vitro/in vivo studies, continuous predictions may be necessary [44,45].

## 5. Conclusions

We performed molecular evolutionary analyses of the *S* genes/S proteins in various SARS-CoV-2 Omicron subvariants. First, we discovered that the Omicron subvariants, including the BA1-5 and XBB subvariants, emerged rapidly from their ancestors in 2021–2022. Next, recombination analysis of the *S* gene between Omicron subvariants BJ.1, BM.1.1.1, and XBB.1.5 showed that the amino acid mutations in the S protein of XBB.1.5 almost completely matched with the parent strains, BJ.1 and BM.1.1.1, suggesting that the XBB.1.5 subvariant was a recombinant between the BJ.1 and BM.1.1.1 subvariants. The genome network analysis suggested that an ancestor of the Omicron BA subvariant formed a genetic network with many amino acid mutations from parental variants such as the Alpha, Beta, and Delta variants. Moreover, we studied the relationships between mutations and conformational epitopes of the S protein, including the SARS-CoV-2 Omicron subvariants XBB.1.5 and XBB.1.16. We also found that most of the mutations in the protein may be associated with their antigenicity and vaccine effects. These mutations were identified as positive selection sites that could counteract host immunity, functioning as escape mutants. Taken together, these mutations may reduce the effectiveness of the current vaccines, including bivalent vaccines such as mRNA vaccines containing the BA.4/BA.5 subvariant sequences. Therefore, the development of a new vaccine may be necessary to target these subvariants.

## Figures and Tables

**Table 1:** Analysis of conformational epitopes on the SARS-CoV-2 mutations. The results of the conformational epitope analysis were compared mutation by mutation, and only regions of concordance were excerpted. Mutations consistent with all three analyses are indicated in bold letters. Mutations consistent with positive selection by selection pressure analysis are shown in red letters, and mutations unique to conformational epitope analysis are shown in blue letters. The areas highlighted in a pale yellow color indicate the RBD regions.

**BA.2**		**BA.4**		**BA.5**	
**Position**	**Predicted epitopes**	**Position**	**Predicted epitopes**	**Position**	**Predicted epitopes**
109–114	**TLDSKT**	108–114	T**TLDSKT**	109–114	**TLDSKT**
144–154	Y**YHKNNKSW**ME	142–152	DVY**YHKNNKSW**	145–152	**YHKNNKSW**
160–169	YSSA**NNCT**FE	164–167	**NNCT**	160–167	YSSA**NNCT**
177–185	MDLEG**KQGN**	182–185	**KQGN**	182–185	**KQGN**
436–452	WNSNKLD**SKVGGNYN**YL	437–450	NSNKLD**SKVGGNYN**	443–450	**SKVGGNYN**
458–460	**KSN**	455–460	LFR**KSN**	457–460	R**KSN**
474–488	**QAGNKPCNGVAGFNC**	468–494	ISTEIY**QAGNKPCNGVAGVNC**YFPLQS	472–489	IY**QAGNKPCNGVAG** **VNC**Y
498–506	**RPTYGVGHQ**	497–506	F**RPTYGVGHQ**	496–506	GF**RPTYGVGHQ**
703–705	**NSV**	703–705	**NSV**	703–705	**NSV**
834–842	IK**QYGDCL**G	836–841	**QYGDCL**	836–841	**QYGDCL**
890–897	**AGAALQ**IP	890–895	**AGAALQ**	890–897	**AGAALQIP**
1140–1162	**PLQPELDSFKEELDKYFKNHTSP**	1140–1162	**PLQPELDSFKEELDKYFKNHTSP**	1140–1162	**PLQPELDSFKEELDKYFKNHTSL**
**BA.4**		**XBB.1.5**		**XBB.1.16**	
**Position**	**Predicted epitopes**	**Position**	**Predicted epitopes**	**Position**	**Predicted epitopes**
142–152	DVYYH**KNNKS**W	146–151	**QKNNKS**	147–151	**KNNKS**
164–167	**NNC**T	164–166	**NNC**	162–166	SA**NNC**
182–185	**KQGN**	182–185	**KEGN**	182–185	**KEGN**
437–450	**NSNKLDSKVGGNYN**	436–452	W**NSNKLDSKPSGNYN**YL	436–452	W**NSNKLDSKPSGNYN**YL
455–460	**LFRKSN**	455–460	**LFRKSK**	455–460	**LFRKSK**
468–494	ISTE**IYQAGNKPCNGVAGVNCYFPLQS**	472–494	**IYQAGNKPCNGVAGP** **NCYSPLQS**	469–494	STE**IYQAGNRPCNGVAGP** **NCYSPLQS**
497–506	**FRPTYGVGHQ**	498–506	**RPTYGVGHQ**	496–506	GF**RPTYGVGHQ**
703–705	**NSV**	703–705	**NSV**	703–705	**NSV**
836–841	**QYGDCL**	836–843	**QYGDCL**GD	834–841	IK**QYGDCL**
890–895	**AGAAL**Q	890–895	**AGAAL**Q	890–894	**AGAAL**
1140–1162	**PLQPELDSFKEELDKYFKNHTSP**	1140–1162	**PLQPELDSFKEELDKYFKNHTSP**	1140–1162	**PLQPELDSFKEELDKYFKNHTSP**
**BA.5**		**XBB.1.5**		**XBB.1.16**	
**Position**	**Predicted epitopes**	**Position**	**Predicted epitopes**	**Position**	**Predicted epitopes**
142–152	YH**KNNKS**W	146–151	**QKNNKS**	147–151	**KNNKS**
160–167	YSS**ANNC**T	164–166	**NNC**	162–166	SA**NNC**
182–185	**KQGN**	182–185	**KEGN**	182–185	**KEGN**
443–450	**SKVGGNYN**	436–452	WNSNKLD**SKPSGNYN**YL	436–452	WNSNKLD**SKPSGNYN**YL
457–460	**RKSN**	455–460	LF**RKSK**	455–460	LF**RKSK**
472–489	**IYQAGNKPCNGVAGV** **NCY**	472–494	**IYQAGNKPCNGVAGP** **NCY**SPLQS	469–494	**STEIYQAGNRPCNGVAGP** **NCY**SPLQS
496–506	GF**RPTYGVGHQ**	498–506	**RPTYGVGHQ**	496–506	GF**RPTYGVGHQ**
703–705	**NSV**	703–705	**NSV**	703–705	**NSV**
836–841	**QYGDCL**	836–843	**QYGDCL**GD	834–841	IK**QYGDCL**
890–897	**AGAALQIP**	890–895	**AGAAL**Q	890–894	**AGAAL**
1140–1162	**PLQPELDSFKEELDKYFKNHTSL**	1140–1162	**PLQPELDSFKEELDKYFKNHTSP**	1140–1162	**PLQPELDSFKEELDKYFKNHTSP**

**Table 2:** Conformational epitope analysis of the SARS-CoV-2 Omicron subvariants BJ.1, BM.1.1.1, XBB.1.5, and XBB.1.16. The results of the conformational epitope analysis were compared for each mutation, and only matched regions were excerpted. The mutated regions are indicated by red letters. Areas highlighted in light yellow indicate RBD regions.

	**BJ.1**		**BM.1.1.1**
**Position**	**Predicted epitopes**	**Position**	**Predicted epitopes**
147–151	**KNNKS**	146–152	H**ENNKSR**
182–185	**KEGN**	182–185	**KQGN**
437–452	NS**NKLDSKPSGNYNYL**	439–452	**NKLDSKVSGNYNYL**
455–460	LF**RKSN**	457–460	**RKSK**
474–489	**QAGNKPCNGAAGFNCY**	472–489	IY**QAGNKPCNGVAGSNCY**
491–506	PLRSYGF**RPTYGVGHQ**	498–506	**RPTYGVGHQ**
703–705	**NSV**	703–705	**NSV**
836–842	**QYGDCL**G	834–841	IK**QYGDCL**
890–896	**AGAAL**QI	890–896	**AGAAL**QI
1140–1162	**PLQPELDSFKEELDKYFKNHTSP**	1140–1162	**PLQPELDSFKEELDKYFKNHTSP**
	**XBB.1.5**		**XBB.1.16**
**Position**	**Predicted epitopes**	**Position**	**Predicted epitopes**
146–151	**QKNNKS**	147–151	**KNNKS**
182–185	**KEGN**	182–185	**KEGN**
436–452	WNS**NKLDSKPSGNYN**YL	436–452	WNS**NKLDSKPSGNYN**YL
455–460	LF**RKSK**	455–460	LF**RKSK**
472–494	IY**QAGNKPCNGVAGPNCY**SPLQS	469–494	STEIY**QAGNRPCNGVAGPNCY**SPLQS
498–506	**RPTYGVGHQ**	496–506	GF**RPTYGVGHQ**
703–705	**NSV**	703–705	**NSV**
836–843	**QYGDCL**GD	834–841	IK**QYGDCL**
890–895	**AGAAL**Q	890–894	**AGAAL**
1140–1162	**PLQPELDSFKEELDKYFKNHTSP**	1140–1162	**PLQPELDSFKEELDKYFKNHTSP**

**Table 3:** Analysis of selection pressures of the SARS-CoV-2 Omicron subvariants. Positive selection pressure assessed using the IFEL method. Major mutations compared to BA.2 are indicated in bold letters.

	**Amino Acid Changes**
	Val3Gly
	Ala83Val
	His146Gln
	**Gln180Glu**
	Glu183Val
	Gly213Glu
	Gly252Val
RBD	Asp339His
	Arg346Thr
	Leu368Ile
	Asn417Lys
	Lys440Asn
	Val445Pro, Ala
	Gly446Ser
	**Leu452Arg**
	**Asn460Lys, Ser**
	**Lys478Arg**
	**Phe486Val, Pro, Ser**
	**Phe490Ser**
	**Arg493Gln**
	Pro521Thr, Gln
	Asn658Ser

## Data Availability

The data presented in this study are available on request from the corresponding author.

## Supplementary Materials

The following supporting information can be downloaded at https://www.mdpi.com/article/10.3390/microorganisms11092336/s1, Figure S1: Conformational epitopes and mutations mapped on the 3D structure models of the S proteins of SARS-CoV-2 variants.
