# Supplementary material for: Molecular Evolutionary Analyses of the Spike Protein Gene and Spike Protein in the SARS-CoV-2 Omicron Subvariants
Source: Microorganisms. 2023 Sep 18;11(9):2336. doi: 10.3390/microorganisms11092336 (PMC10537508; doi:10.3390/microorganisms11092336)
Supplement: Supplementary file 1 [file microorganisms-11-02336-s001.zip › microorganisms-2555777-supplementary.pdf]

	Protein	Predicted epitopes
BA.2	14-19	QCVNLR
	109114	TLDSKT
	144-154	YYHKNNKSWME
	160-169	YSSANNCTFE
	177-185	MDLEGKQGN
	436-452	WNSNKLDSKVGGNYNYL
	458-460	KSN
	474-488	QAGNKPCNGVAGFNC
	493-496	RSYG
	498-506	RPTYGVGHQ
	553-563	TESNKKFLPFQ
	582-584	LEI
	703-705	NSV
	809-812	PSKP
	834-842	IKQYGDCLG
	890-897	AGAALQIP
	912-914	TQN
	917-919	YEN
	1140-1162	PLQPELDSFKEELDKYFKNHTSP
BA.2.10	15-17	CVN
	146-151	HKNNKS
	180-185	EGKQGN
	343-346	NATR
	415-417	TGN
	436-452	WNSNKLDSKVGGNYNYL
	455-460	LFRKSN
	468-472	ISTEI
	474-488	QAGNKPCNGVAGFNC
	490-496	FPLRSYG
	498-506	RPTYGVGHQ
	553-563	TESNKKFLPFQ
	703-705	NSV
	809-812	PSKP
	834-843	IKQYGDCLGD
	890-895	AGAALQ
	1140-1162	PLQPELDSFKEELDKYFKNHTSP
BA.2.75	146-151	HENNKS
	172-175	SQPE
	180-185	EGKQGN
	343-346	NATR
	415-417	YGN
	436-452	WNSNKLDSKVSGNYNYL
	455-460	LFRKSK
	468-506	ISTEIYQAGNKPCNGVAGFN CYFPLQSYGFRPTYGVGHQ
	555-561	SNKKFLP
	703-705	NSV
	808-813	DPSKPS
	836-842	QYGDCLG
	890-895	AGAALQ
	912-914	TQN
	917-919	YEN
	1070-1074	AQEKN
	1140-1162	PLQPELDSFKEELDKYFKNHTSP

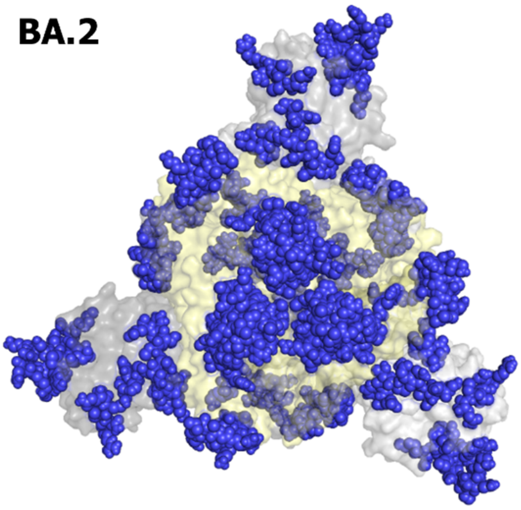

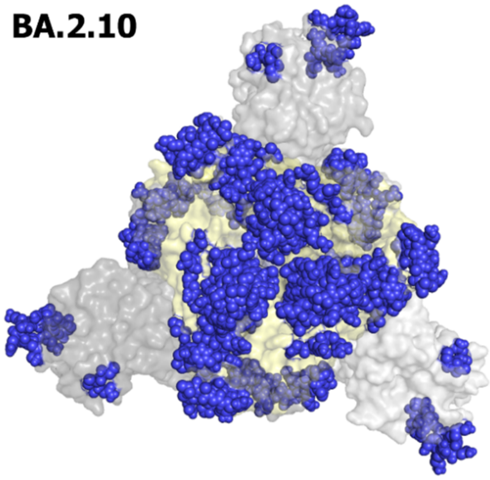

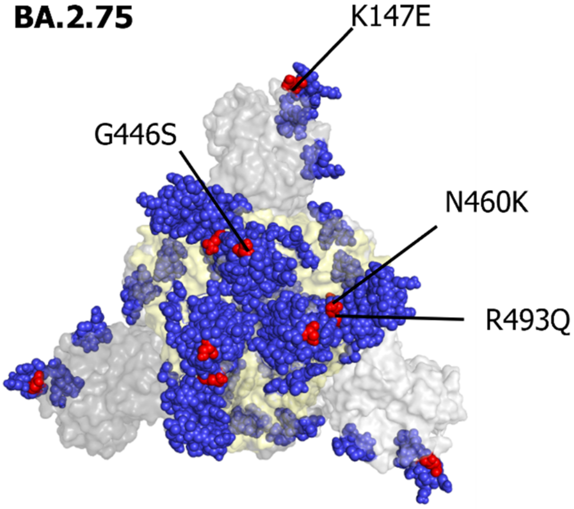

BA.4	108-114	TTLDSKT	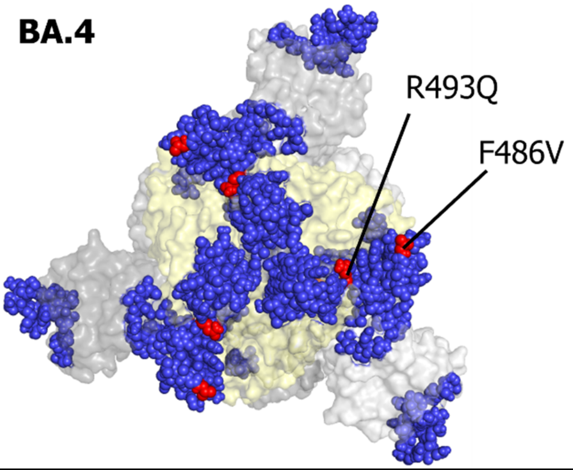
	142-152	DVYYHKNNKSW	
	164-167	NNCT	
	182-185	KQGN	
	254-256	SSS	
	415-417	TGN	
	437-450	NSNKLDSKVGGNYN	
	455-460	LFRKSN	
	468-494	ISTEIYQAGNKPCNGVAGV NCYFPLQS	
	497-506	FRPTYGVGHQ	
	703-705	NSV	
	836-841	QYGDCL	
	890-895	AGAALQ	
	1140-1162	PLQPELDSFKEELDKYFKNHTSP	
BA.5	14-17	QCVN	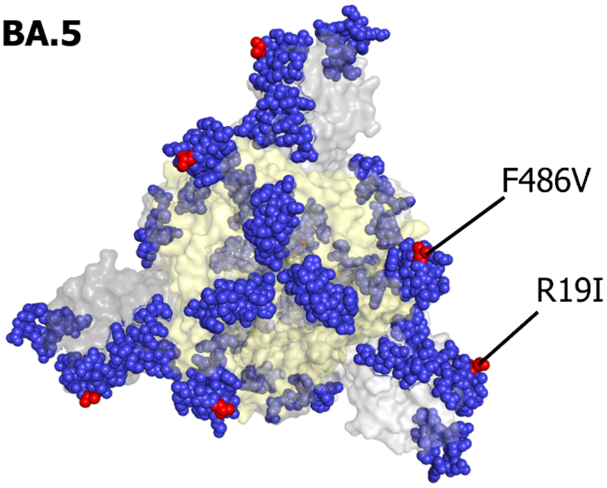
	19-21	ITR	
	109-114	TLDSKT	
	134-137	QFCN	
	145-152	YHKNNKSW	
	160-167	YSSANNCT	
	182-185	KQGN	
	254-259	SSSGWT	
	443-450	SKVGGNYN	
	457-460	RKSN	
	472-489	IYQAGNKPCNGVAGVNCY	
	496-506	GFRPTYGVGHQ	
	554-561	ESNKKFLP	
	703-705	NSV	
	809-812	PSKP	
	836-841	QYGDCL	
	890-897	AGAALQIP	
	912-914	TQN	
	917-919	YEN	
	1070-1074	AQEKN	
	1140-1162	PLQPELDSFKEELDKYFKNHTSL	
BJ.1	147-151	KNNKS	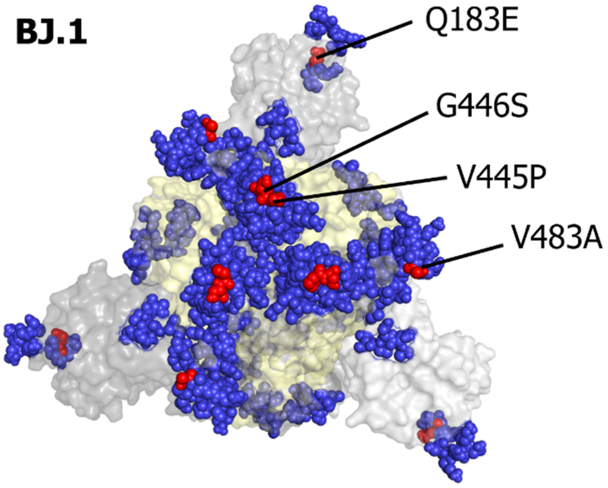
	163-167	ANNCT	
	182-185	KEGN	
	415-417	TGN	
	437-452	NSNKLDSKPSGNYNYL	
	455-460	LFRKSN	
	468-472	ISTEI	
	474-489	QAGNKPCNGAAGFNCY	
	491-506	PLRSYGFRPTYGVGHQ	
	703-705	NSV	
	809-812	PSKP	
	836-842	QYGDCLG	
	890-896	AGAALQI	
	1140-1162	PLQPELDSFKEELDKYFKNHTSP	

BM.1.1.1	146-152	HENNKSR
	182-185	KQGN
	211-214	NLGR
	439-452	NKLDSKVSGNYNYL
	457-460	RKSK
	472-489	IYQAGNKPCNGVAGSNCY
	491-496	PLQSYG
	498-506	RPTYGVGHQ
	555-563	SNKKFLPFQ
	703-705	NSV
	809-812	PSKP
	834-841	IKQYGDCL
	885-887	GWT
	890-896	AGAALQI
	912-914	TQN
	1070-1074	AQEKN
	1140-1162	PLQPELDSFKEELDKYFKNHTSP
XBB.1.5	16-20	VNLRT
	111-114	DSKT
	146-151	QKNNKS
	164-166	NNC
	182-185	KEGN
	255-258	SSGW
	343-346	NATT
	436-452	WNSNKLDSKPSGNYNYL
	455-460	LFRKSK
	472-494	IYQAGNKPCNGVAGPNCYSPLQS
	498-506	RPTYGVGHQ
	703-705	NSV
	836-843	QYGDCLGD
	890-895	AGAALQ
	1140-1162	PLQPELDSFKEELDKYFKNHTSP
XBB.1.16	147-151	KNNKS
	162-166	SANNC
	182-185	KEGN
	343-346	NATT
	414-417	QTGN
	436-452	WNSNKLDSKPSGNYNYL
	455-460	LFRKSK
	469-494	STEIYQAGNRPCNGVAGPNCYSPLQS
	496-503	GFRPTYGVGHQ
	556-558	NKK
	703-705	NSV
	809-812	PSKP
	834-841	IKQYGDCL
	890-894	AGAAL
	912-914	TQN
	917-919	YEN
	1140-1162	PLQPELDSFKEELDKYFKNHTSP

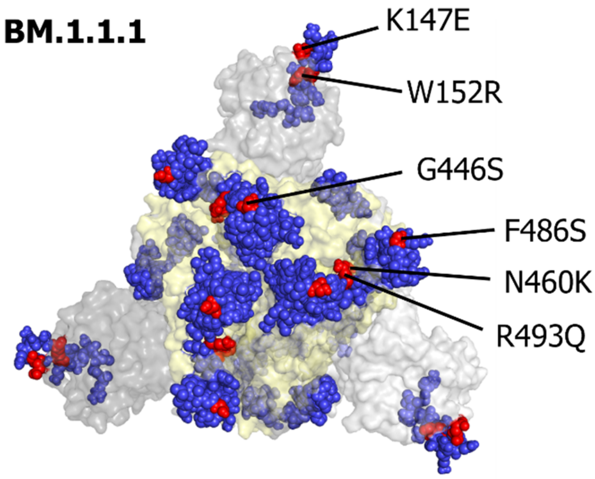

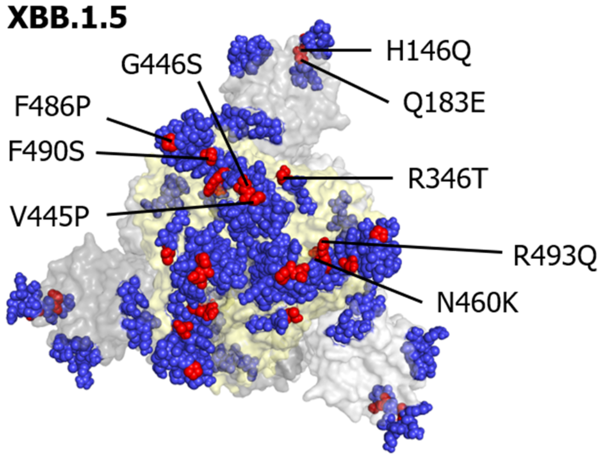

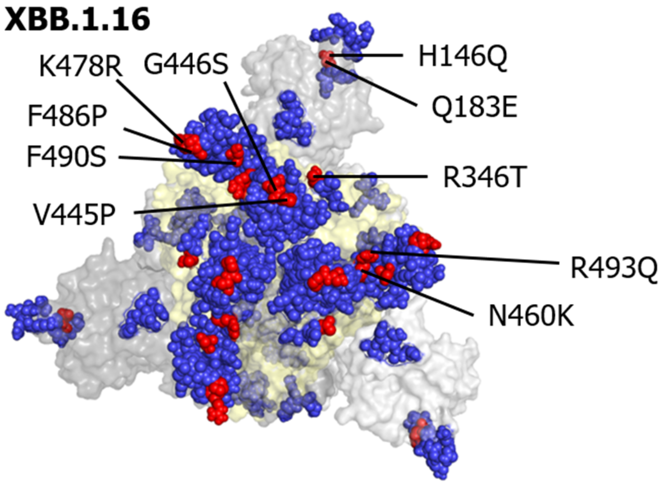

Figure S1. Conformational epitopes and mutation mapped on three-dimensional structure models of the SARS-CoV-2 variants in the S protein. The results of the conformational epitope analysis of the SARS-CoV-2 mutant and the mapping of the predicted conformational epitopes and mutation sites on the conformational model of the SARS-CoV-2 S protein are shown. Amino acids mutated from BA.2 on the predicted epitope are indicated in red letters. The chains of the trimeric structure are color-coded in dark gray (chain A), gray (chain B), and light gray (chain C). Conformational epitopes, mutations on conformational epitopes, and RBDs are shown in blue, red, and light yellow, respectively.
